# Placebos in Healthcare: A Behavioral Study on How Treatment Responsiveness Affects Therapy Decisions in a Simulated Patient–Physician Interaction

**DOI:** 10.3390/clinpract14050170

**Published:** 2024-10-17

**Authors:** Alessandro Piedimonte, Valeria Volpino, Francesco Campaci, Francesca Borghesi, Giulia Guerra, Elisa Carlino

**Affiliations:** 1 Department of Neuroscience “Rita Levi Montalcini”, University of Turin, Corso Raffaello 30, 10125 Turin, Italy; 2 Carlo Molo Foundation Onlus, Via della Rocca 24bis, 10123 Turin, Italy; 3 Department of Psychology, University of Turin, Via Verdi 8, 10124 Turin, Italy

**Keywords:** general practice, treatment outcome, physician–patient relationship, placebos, placebo effect, empathy

## Abstract

Background and Purpose: Treatment choice during clinical practice is crucial to best help each patient. One of the physicians’ main goals is choosing a personalized effective treatment, but it also represents a challenging issue. Here, we explored different treatment choices in a simulated patient–physician interaction. Materials and Methods: Medical students (*n* = 48) and young Practicing Physicians (*n* = 20) were recruited to behave as “physicians” while fellow researchers acted as “patients”. Participants were divided equally into a Belief Group, which received positive information about placebo efficacy, and a Non-Belief Group, which received negative information. Empathy traits and psychological variables were measured in both groups. During the task, participants were asked to choose between an active (TENS treatment) or a placebo treatment, to reduce patients’ pain. Patients never underwent the painful stimulation but acted as if they had, simulating high or low pain responses to the placebo treatment (placebo-responders/placebo non-responders) and low pain to the TENS treatment. Results: Behavioral results showed that the Belief Group gave significantly more placebo treatments when faced with a patient that simulated placebo responsiveness, while the Non-Belief group showed a mirrorlike behavior, administrating more believed TENS treatments when faced with a placebo non-responder. No differences were found between Medical Students and Practicing Physicians. Conclusions: This study constitutes a frame of reference for medical treatment decisions, indicating that physicians’ treatment choices are influenced by patients’ responsiveness to the treatments, as well as by their prior beliefs and empathy traits.

## 1. Introduction

Recently, Garcia-Larrea and Bouhassira disputed general pain medical practice since it blindly follows the utilitarianism motto “the greatest good for the greatest number”; they argued that the treatment administered to a patient is the statistically accepted one, proving to be valid for the population. However, as they pointed out, medicine too often forgets the uniqueness of patients and their responses to a particular treatment [1] Generally, placebos are defined as inert treatments without active principles that are administered with different aims [2]) and in various contexts. In clinical trials, placebos are given to test the efficacy of active treatment compared to the efficacy of inert treatments (the so-called “placebo response”, following the gold standard of randomized double-blind placebo-controlled clinical trials (RCTs)) [3,4]. In experimental settings, placebos are used to study the effect of the administration of an inert treatment in a psychosocial context made of cues and rituals; this is the so-called “placebo effect”, which is triggered by specific and well-documented psychological mechanisms and produces a cascade of neurobiological events [2,5,6,7,8,9,10]. Finally, in clinical practice, especially in tertiary care and among nursing students [11,12,13] (the use of placebo treatments can be ethically, professionally, and legally controversial [14]), and it comes with the problem of whether and how to communicate this practice to the patient, with a wide variety of views and “opinions” reported [12]. On top of this, different placebos can be administered: the so-called impure placebos and pure placebos. The use of impure placebos (i.e., pharmacologically active agents with intrinsic therapeutic effects for other diseases than the one the patient is suffering) is less employed compared with the administration of pure placebos (i.e., treatments without pharmacological effects) [12,15].

Despite any concerns, placebos can be considered powerful tools to increase the pharmacological effect of real treatments [5,16,17] or remedies used when no active treatments are available. Undoubtedly, placebo responders exist, and the placebo effect can have a high magnitude, as reported in the results of many RCTs and meta-analyses [18,19,20,21,22]. Moreover, ethical problems may be partially overcome with the prescription of placebos in full transparency (Open Label Placebo, OPL), as recent studies show the possibility of observing placebo hypoalgesia without deception [23,24]. 

However, despite the increased literature about the effects of placebo administrations on the patient’s brain and the well-known attitude of administering placebo interventions in clinical practice, to our knowledge, no studies to date have investigated the figure of the “placebo giver” into a scientific laboratory setting; here, the “placebo giver” is conceptualized as the clinician that, deliberately or not, administers a placebo during a clinical intervention.

In this study, using simulated patient–physician interactions, we investigated Medical Students and young Practicing Physicians who actively deliver interventions to reduce pain. In a simulated patient–physician relationship, we studied the factors that can influence a physician in prescribing one of two different treatments. To do so, participants were asked to ameliorate experimentally induced pain; the painful stimulation was delivered to other healthy individuals, who were fellow researchers instructed by the experimenters to behave according to our experimental design. Participants could choose between two Transcutaneous Electrical Nerve Stimulation (TENS) treatments, both of which were actually inactive. Participants believed that one treatment was active (from now referred to as “believed TENS treatment”) and knew that the other one was a placebo/the inactive one (from now referred to as “placebo treatment”). In both cases, TENS treatments were inactive. Two different variables were considered and manipulated, as follows: (1) cognitive variables, namely the knowledge of the participants about the efficacy of the placebo treatment (Belief Group vs. Non-Belief Group); and (2) the subjective feedback provided by the patients about the effectiveness of treatments (high vs. low painful responses). Finally, psychological variables such as empathy, cognitive flexibility, skepticism, and self-efficacy were taken into account. We sought to understand how personalized pain management may be patient-centered and guided; specifically, here it is explored if, and under which conditions, a physician may choose to administer a placebo treatment instead of an active one.

## 2. Materials and Methods

### 2.1. Participants

A total of 48 healthy volunteers (23 males, 25 females, age = 21.23 ± 1.08) were recruited among the students at the Medicine University of Turin and were engaged in the study after signing a written informed consent form. The inclusion criteria required that participants, referred to as “Medical Students”, were students of the medical school and enrolled in the first 3 years of the medicine education training. Medical Students were informed that they would take part in a study investigating pain perception in a special patient–physician context, in which they had to choose a treatment (believed TENS treatment or placebo treatment) to reduce pain induced by other participants (actors recruited by the experimenters). Moreover, a total of 20 healthy volunteers (11 males, 9 females, age = 30.6 ± 1.7) were additionally enrolled in the study as “Young Practicing Physicians”. The inclusion criteria required that participants, referred to as “Practicing Physicians”, were physicians with experience of fewer than 5 years. In our population of Practicing Physicians, 12 were continuity of care physicians and 8 were primary care physicians. Both of these groups will be collectively referred to as “Participants” to differentiate them from “Patients”.

Two 23-year-old confederates were trained to play the “Patients” according to a rehearsed script described before the experiment. They were both Caucasian and similar in demographic, social, and personality aspects.

### 2.2. Experimental Procedure

Participants were introduced to the experimental procedure by reading the information included in the informed consent and listening to instructions given by the experimenters. They were informed that the study aimed to investigate pain perception in a simulated patient–physician context, in which they had to act as the physicians and decide the appropriate treatment (believed TENS treatment or placebo treatment) to reduce pain in two different patients. Before the experiment, participants were asked to rate their knowledge of the placebo phenomenon; in particular, they were asked to rate, from 0 to 10, how much they were informed about the effectiveness of a placebo treatment in a pain management procedure. After the experiment, they were asked to rate on a scale from 0 to 10 about their belief in placebo treatments.

They were given the following information: “Today we will simulate a patient-physician interaction, you are the physician and another volunteer will be the patient. The patient will lie on this bed and will receive 80 electrical stimuli on the dorsum of his/her hand. The intensity of the stimuli varies based on the subjective pain threshold of each patient. The electrical stimuli induce painful sensations that range from 0 (no pain) to 10 (unbearable pain): stimuli rated from 0 to 4 are low painful stimuli, whereas stimuli rated from 7 to 10 are high painful stimuli. During this experiment, you are asked to act as a physician in a real patient-physician interaction: thus, you are asked to choose the treatment for the patient to reduce his/her pain. After the first electrical stimulation, the patient will verbally rate his/her pain and then you will have time to choose and deliver the analgesic treatment; after that, a second stimulation will be delivered along with the treatment you have chosen and the patient will rate again the pain intensity. With this second rating, you will know if the treatment delivered has been effective in reducing pain. You can choose between two different treatments: a real treatment (believe TENS treatment) or a placebo treatment. Since pain is well localized and induced by a controlled electrical stimulator, the real treatment consists of the activation of two TENS electrodes that block the painful transmission, as postulated by the gate control theory of pain. You can activate these electrodes by pressing the red button of the electrical stimulator. The placebo treatment is a sham activation of the electrodes and can be delivered by pressing the black button of the electrical stimulator. You can write the pain ratings of the patients on a paper and you can also take a record of the treatments you decide to deliver”.

**Figure 1:**
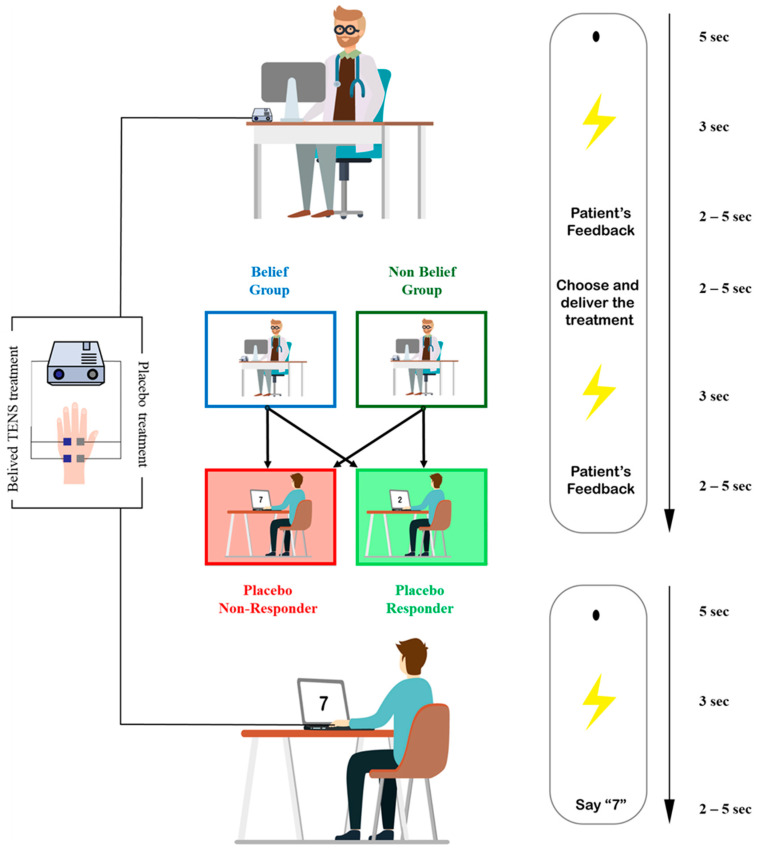
Experimental paradigm. Experimental timeline and representation of the experimental groups.

Participants were also informed about the duration of each trial, presented on a computer screen positioned in front of them (Figure 1). The sequence started with a resting period of 5 s, then a light appeared on a computer screen indicating the start of the electrical stimulation. At the end of the stimulation, patients had 2 to 5 s to rate the intensity of the pain they experienced while the text “Patient’s Feedback” was displayed. Then, the text “Choose and Deliver the Treatment” appeared on the physician’s computer screen, signaling they had to make a choice on which treatment they preferred and deliver it. Then, a second light appeared indicating the second electrical stimulation, followed again by the rating procedure. Participants were also asked to write down the rating of the patients and the treatment chosen. 

### 2.3. Cognitive Manipulation

After listening to the description of the experimental procedure, participants were randomly allocated to two different groups, the Belief Group or the Non-Belief Group, based on the cognitive information provided by the experimenters. Participants in the Belief Group were informed about the effectiveness of a placebo intervention, whereas participants in the Non-Belief Group were informed about the lack of effectiveness of the placebo intervention.

The following instructions were delivered to the Belief Group: “As described before, the real treatment is effective in reducing pain, as two electrodes are activated to block the painful stimuli closing the gate and reducing pain. This stimulation can produce side effects, such as redness of the skin. On the other hand, the placebo treatment is a sham procedure that doesn’t activate the TENS electrodes. As extensively documented by the literature on pain, placebo interventions are often effective against pain [6,25]. Patients are not aware of the possibility of receiving verum or placebo treatment, as they believe that only a verum treatment will be administered. Thus, you can freely choose the treatment to deliver. Again, consider that both treatments are effective in reducing pain but the placebo treatment is a sham and for this reason, it doesn’t produce redness of the skin as a side effect”.

The following instructions were delivered to the Non-Belief Group: “As described before, the real treatment is effective in reducing pain, as two electrodes are activated to block the painful stimuli closing the gate and reducing pain. This stimulation can produce side effects, such as redness of the skin. On the other hand, the placebo treatment is a sham procedure that doesn’t activate the TENS electrodes. As extensively documented by the literature on pain, placebo interventions are often not effective against pain [26]. Patients are not aware of the possibility of receiving verum or placebo treatment, as they believe that only a verum treatment will be administered. Thus, you can freely choose the treatment to deliver. Again, consider that the real treatment can produce redness of the skin as a side effect but has an analgesic effect, whereas the effectiveness of placebo treatments is based on subjective patients’ expectations. This means that the placebo effect is more variable and its magnitude is reduced compared to the verum treatment effect”.

Following the cognitive manipulation on whether the placebo treatment could have positive or negative outcomes, participants were introduced to the first patient while the experimenter applied four electrodes (two electrodes for the electrical stimuli and two for the treatment) on the dorsum of the patient’s right hand. Patients were asked to rate their pain intensity using a scale from 0 to 10 before and after the administration of the treatment. They were asked to look at the computer screen to see the beginning of the electrical stimulation and the timing of the treatment’s administration; actually, no electrical stimuli were delivered, and patients looked at the computer screen just to read the pain ratings they had to report to simulate alleged placebo responsiveness or non-responsiveness. After 80 fake stimulations, meaning 40 participants’ treatment choices, the experiment ended and the second patient entered the room.

### 2.4. Feedback Manipulation

The feedback participants received was manipulated; in fact, patients read pain ratings on the computer screen, not visible to physicians, and were asked to report them without any facial or emotional involvement, to standardize the experimental procedure. The feedback was manipulated as follows: when the participants chose to administrate the believed TENS treatment, only low pain ratings were reported (ranging from 2 to 4), to simulate good responsiveness to the active treatment. When the participants chose the placebo treatment, patients had to report low pain ratings (ranging from 2 to 4) when they simulated placebo responsiveness; meanwhile, patients had to report high pain ratings (ranging from 7 to 9) if they simulated placebo non-responsiveness. In this way, participants interacted with two different kinds of patients. In a random order, one simulated a placebo responsiveness and the other simulated a placebo non-responsiveness, whereas both always simulated a high responsiveness to the believed TENS treatment.

### 2.5. Psychological Variables

Before the experiment, participants were asked to fill out four psychological texts: (1) the Cognitive Flexibility Scale (CF Scale) [27]; (2) the Jefferson Scale of Physician Empathy (JSPE Scale) [28]; (3) the Skepticism Scale (SS Scale) [29], and (4) the General Self-Efficacy Scale (GSE Scale) [30]. Moreover, participants were asked to rate their knowledge about the placebo phenomenon on a scale from 0 (no knowledge) to 10 (expert knowledge). This rating was asked before and after the cognitive manipulation.

### 2.6. Statistical Analysis

For each group (Belief Group and Non-Belief Group in both cohorts: Medical Students and Practicing Physicians), the Wilcoxon test for matched pairs was used to check for differences between placebo and believed TENS treatments administered to placebo responders and non-responders. Furthermore, correlations between all psychological tests (CF, JSPE, SS, GSE) and the number of placebo treatments administered were performed.

Furthermore, the Mann–Whitney U test was used to assess the differences between the number of placebo treatments administered by the two groups (Belief Group and Non-Belief Group in both cohorts: Medical Students and Practicing Physicians), when they were faced with either the placebo responders or the placebo non-responders.

The Mann–Whitney U test was also carried out to assess differences between the two cohorts, Medical Students and Practicing Physicians.

Statistical analyses have been conducted using StatSoft STATISTICA software (v10; www.statsoft.com (accessed on 8 May 2023)).

## 3. Results

Participants (Medical Students and Practicing Physicians) who were assigned to the Belief Group and the Non-Belief Group were comparable in terms of their demographic characteristics. Medical Students and Practicing Physicians differed in their knowledge of the placebo phenomenon and its effects (t = 12.32; *p* < 0.001): Medical Students reported a mean knowledge of 1.25 and Practicing Physicians reported a mean knowledge of 4.15 on a scale from 0 to 10.

### 3.1. Belief Group

**Figure 2:**
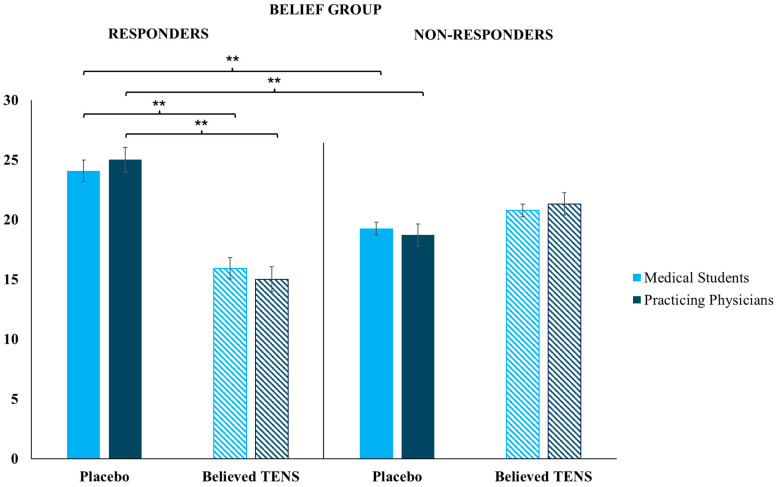
Belief Group results. Participants in the Belief Group (Medical Students and Practicing Physicians) administered significantly more placebo treatments in comparison with the believed TENS treatments when faced with placebo responsiveness; participants in the Belief Group (Medical Students and Practicing Physicians) administered significantly more placebo treatments when the patient was a placebo responder than when they were a non-responder. Error bars represent the standard error of the mean (SEM); ** *p* < 0.01.

Results are outlined in Figure 2. In the cohort of Medical Students, when the Belief Group was faced with placebo responders, participants administered significantly more placebo treatments in comparison to believed TENS treatments (Z = 3.5, *p* < 0.001), while there was no significant difference between placebo and believed TENS treatments administered when this group was faced with placebo non-responders. To confirm this result, in the Belief Group, significantly more placebo treatments (i.e., significantly fewer believed TENS treatments) were administered when a physician from this group met a placebo responder in comparison to a non-responder (Z = 3.6, *p* < 0.001). Similarly, in the cohort of Practicing Physicians, when the Belief Group was faced with placebo responders, they administered significantly more placebo treatments in comparison to believed TENS treatments (Z = 2.8, *p* < 0.01), while there was no significant difference between placebo and believed TENS treatments administered when this group was faced with placebo non-responders. Again, to confirm this result, in the Belief Group, significantly more placebo treatments (i.e., significantly fewer believed TENS treatments) were administered when a physician from this group met a placebo responder in comparison to a non-responder (Z = 2.8, *p* < 0.001).

### 3.2. Non-Belief Group

**Figure 3:**
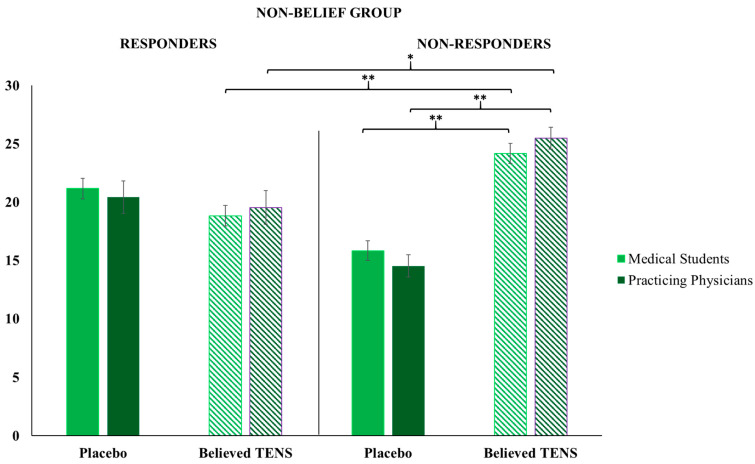
Non-Belief Group results. Participants in the Non-Belief Group (Medical Students and Practicing Physicians) administered significantly more believed TENS treatments than placebos when faced with placebo non-responders; participants in the Non-Belief Group (Medical Students and Practicing Physicians) administered significantly more believed TENS treatments when the patient was a placebo non-responder, than when they were a responder. Error bars represent the standard error of the mean (SEM); * *p* < 0.05, ** *p* < 0.01.

Results are outlined in Figure 3. In the cohort of Medical Students when the Non-Belief Group was faced with placebo responders, no significant difference between placebo and believed TENS treatments administered was observed, while when this group was faced with placebo non-responders, participants administered significantly more believed TENS treatments in comparison to placebo treatments (Z = 3.9, *p* < 0.001). Again, in the Non-Belief Group, significantly more believed TENS treatments (i.e., significantly fewer placebo treatments) were administered to placebo non-responders in comparison to responders (Z = 3.4, *p* < 0.001). Similarly, in the cohort of Practicing Physicians, when the Non-Belief Group was faced with placebo responders, no significant difference between placebo and believed TENS treatments administered was observed, while when this group was faced with placebo non-responders, participants administered significantly more believed TENS treatments in comparison to placebo treatments (Z = 2.8, *p* < 0.01). Again, in the Non-Belief Group, significantly more believed TENS treatments (i.e., significantly fewer placebo treatments) were administered to placebo non-responders in comparison to responders (Z = 2.2, *p* < 0.03).

Furthermore, in the cohort of Medical Students, the Mann–Whitney U test showed that Belief Group participants administered significantly more placebo (i.e., less believed TENS treatments) treatments in comparison with Non-Belief Group participants when faced either with placebo responders (Z_adj_ = 2.5, *p* < 0.05) or non-responders (Z_adj_ = 2.9, *p* < 0.01); symmetrically, Non-Belief Group participants administered significantly more believed TENS treatments (i.e., less placebo) treatments in comparison with Belief Group physicians when faced either with placebo responders (Z_adj_ = 2.5, *p* < 0.05) or non-responders (Z_adj_ = 2.9, *p* < 0.01).

Similarly, the Mann–Whitney U test in the cohort of Practicing Physicians showed that Belief Group physicians administered significantly more placebo (i.e., less believed TENS treatments) treatments in comparison to the Non-Belief Group physicians when faced either with placebo responders (Z_adj_ = 2.1, *p* < 0.05) or non-responders (Z_adj_ = 2.7, *p* < 0.01); symmetrically, Non-Belief Group physicians administered significantly more believed TENS treatments (i.e., less placebo) in comparison with Belief Group physicians when faced either with placebo non-responders (Z_adj_ = 2.7, *p* < 0.01) or responders (Z_adj_ = 2.1, *p* < 0.05).

For overall differences between the two cohorts (Medical Students and Practicing Physicians), the Mann–Whitney U test showed that in the Belief Group, there were no significant differences in the administration of placebo treatments or believed TENS treatments (Z_adj_ = 0.91, *p* > 0.05) when faced with placebo responders, as well as no significant differences in the administration of placebo treatments or believed TENS treatments (Z_adj_ = 0.42, *p* > 0.05) when faced with placebo non-responders. Identically, in the Non-Belief Group, there were no significant differences in the administration of placebo treatments or believed TENS treatments (Z_adj_ = 0.85, *p* > 0.05) when faced with placebo responders, as well as when faced with non-responders (Z_adj_ = 1.63, *p* > 0.05).

### 3.3. Correlation Analysis

**Figure 4:**
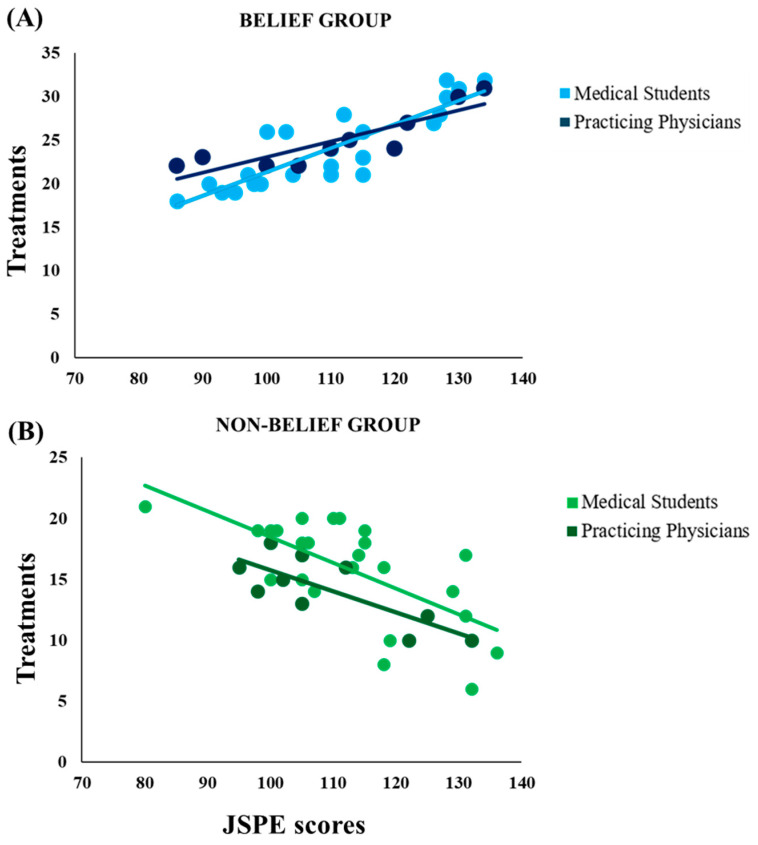
Significant correlations. (**A**) Positive correlation between JSPE scores (*x*-Axis) and number of placebos administered (*y*-Axis) in the Belief Group, both for the Medical Students (r = 0.85, *p* < 0.001) and for the Practicing Physicians (r = 0.87, *p* < 0.01); (**B**) negative correlation (r = 0.67, *p* < 0.001) between JSPE scores (*x* Axis) and number of placebos administered (*y* Axis) in the Non-Belief Group, both for the Medical Students (r = −0.67, *p* < 0.001) and for the Practicing Physicians (r = −0.78, *p* < 0.01).

Correlation analysis on psychological tests in the cohort of Medical Students showed that JSPE scores in the Belief Group positively correlated with the number of placebo treatments administered, but only when participants were faced with placebo responders (r = 0.85, *p* < 0.001) (Figure 4A). In the Non-Belief Group, JSPE scores negatively correlated with the number of placebo treatments administered but only when participants were faced with placebo non-responders (r = −0.67, *p* < 0.001) (Figure 4B). The other psychological test scores (SS, CF, GSE) did not correlate with any of our variables. Finally, correlation analysis on psychological tests in the cohort of Practicing Physicians showed that JSPE scores in the Belief Group positively correlated with the number of placebo treatments administered but only when participants were faced with placebo responders (r = 0.87, *p* < 0.01) (Figure 4A). In the Non-Belief Group, JSPE scores negatively correlated with the number of placebo treatments administered but only when participants were faced with placebo non-responders (r = −0.78, *p* < 0.01) (Figure 4B). The other psychological test scores (SS, CF, GSE) did not correlate with any of our variables.

## 4. Discussion

Great efforts are being made in research and clinical practice to find treatments as effective as possible with few adverse effects, and, recently, personalized pain management has again gathered attention [1]. In the present study, we investigated participants (Medical Student and Practicing Physicians) who needed to choose an intervention for their patients, who exhibited experimental-induced pain manifestations. Two sources of information were manipulated by the experimenters: the previous beliefs of participants and the patients’ feedback on pain intensity. Specifically, we explored if and under which conditions a physician may choose to administer a placebo treatment instead of an active one (believed TENS treatment).

Results showed that patient responsiveness to the treatment received, and believed to be effective by a physician, is crucial for consequential therapy choices. Only participants educated about the positive effects of placebos (Belief Group) administered significantly more placebos compared with the believed TENS treatments, and this happened only when they were facing placebo responders (i.e., a patient (fellow researcher) who gave low pain ratings after receiving a placebo intervention); when participants were facing patients who showed high pain ratings after a placebo (i.e., placebo non-responders), there was no significant differences between the number of placebos administered in respect to the number of believed TENS treatments.

In the Non-Belief Group, in which participants were educated about the lack of efficacy of placebo interventions, we found specular results: participants administered significantly more believed TENS treatments compared with placebos, but only when they were faced with a placebo non-responder. This is relevant: when the Non-Belief Group faced patients who acted as placebo responders, there was no significant difference between the number of placebos administered compared with the believed TENS treatments. This result shows that when having low beliefs in a placebo treatment, and seeing its poor efficacy in practice, the active treatment is the obvious choice, but when no differences are evident between two different treatments (even if one is believed to be not effective) participants did not have a preference in the administration. In clinical practice, physicians could try new ways of treatment when this appears to ameliorate the patient’s state, even if they previously thought the treatment was not effective. In particular, placebos could be used in practice as additive treatments to active ones, as boosters of the psychobiological context around the patient, the therapy, and the clinical relationship itself [31]. For instance, describing basic mechanisms behind the treatment, stating confidently that the treatment is effective, and using an empathic communication style to relate with patients, are all common recommended guidelines that can boost the additive placebo effect, reducing stress and anxiety while increasing hope and modulating expectations [31,32].

In general, results highlight that when participants’ prior knowledge matches with the clinical outcome expected, there is a significantly high chance to administer the same treatment again, but only if the other available treatments are ineffective. Indeed, if both available treatments appear to be effective for the patient, the physicians do not blindly follow their preferred therapy but explore other potential therapeutic solutions. Instead, if the clinical outcome does not match with positive prior beliefs, the chance of administering the same treatment decreases to the advantage of the other available treatment; indeed, there is a significant difference between the number of administrations of treatment believed to be effective (for the Belief Group, placebos) to a responsive patient compared with the number of administrations of the same treatment to a non-responsive patient. In this study, the indirect active role played by the patient during the treatment choice has emerged.

Interestingly, significant correlations were observed between empathy scores and the number of placebo treatments administered in both groups, suggesting a relationship between clinicians’ empathy and the relevance patients have in the therapeutic process. This relationship was observed only when prior knowledge matched with the clinical outcome: in the Belief Group, empathy scores were positively correlated to the number of placebo treatments given but only when participants faced placebo responders; in the Non-Belief Group, empathy scores were negatively correlated to the number of placebo treatments given, but only when participants faced placebo non-responders.

In the current experiment, it seems likely that empathy was “enabled” only when prior knowledge matched the actual context during the experimental task, i.e., only when participants could frame the situation they were in, referring it to something they knew. The result is in line with the conceptualization of empathy as the outcome of a simulation process [33] where both affect sharing [34] and active perspective-taking [35] are involved. In other words, empathy is the result of bottom-up sensorial inputs unified with top-down cognitive expectancies [36] which then shapes behavior. The overall process observed in the experiment is arguable under the predictive coding perspective. Briefly summarizing the theory, perceptions and actions are built upon specific top-down expectations and bottom-up sensory signals. In this specific case, top-down expectations represented the idea that a treatment will have a positive effect on the painful condition, whereas bottom-up sensory signals are the actual patients’feedback. It is important to underline that, in the model of predictive coding, precision is crucial [37]. In this study, the cognitive and patients’ feedback manipulation created a situation of precise match or mismatch between participants’ knowledge (or top-down prediction) and clinical occurrence (or bottom-up sensory signals); i.e., in the first case, the Belief Group with placebo responders and the Non-Belief Group with placebo non-responders, and in the second case, a placebo non-responder for the Belief Group and a placebo responder for the Non-Belief Group. This last scenario led to a prediction error causing the reduction in the differences between the number of placebos and active treatments administered, but it confounded participants about the context expected. In the first case, that is, the presence of a non-responder for the Belief Group, physicians may not clearly understand why personal beliefs do not help in the clinical situation; in the second case, that is, the presence of a responder for the Non-Belief Group, physicians may choose to explore the other effective treatment to ameliorate patients’ condition at best. In the other two scenarios, the match between top-down and bottom-up signals led to a higher probability of maintaining a treatment administration approach consistent with their beliefs and prior choices. In summary, high empathy scores in the JSPE seemed to indicate a higher probability of following previous knowledge when the efficacy of the treatment was confirmed; in other words, higher empathy is associated with a higher sensitivity to bottom-up confirmation of top-down expectations. On the contrary, the lower the empathy, the less capable the positive bottom-up signals are in confirming previous choices. It is plausible that contextual information related to observed individual circumstances influences the empathic process [33] and, consequentially, the behavior.

Overall, this study demonstrates that physician practice is not restricted to a one-way best protocol treatment and it shows its flexibility to patients’ present condition. This result is in line with previous research on psychologists’ practice, in which professionals appeared to take into account their insight and beliefs during the therapy [38], shaping treatments step-by-step to provide the best personalized treatment. In psychotherapy practice, the approach called Process Based Therapy (PBT) [39] is gaining more and more attention from professionals; to summarize the underlying concept is to provide individualized, contextually specific, treatment in a way that is evidence-based, reassessing the therapeutic process step by step and shaping treatment along with patient’s present necessities [40].

As reported in the introduction, placebo responders exist and the placebo effect may have a high magnitude [4,18,19,20,21,22]. Hypothesizing to utilize them in the clinical practice with placebo responders could mean that other forms of treatments, which might have potentially adverse effects, could be avoided, ending up providing greater help to the patient. For instance, RCTs conducted on patients affected by chronic low back pain showed how the administration of open-label placebos (OLP) for three consecutive weeks reduced reported pain ratings and pain-related disability by 30% compared to baseline levels, while the group treated with their usual pain medication reported a reduction in pain of 9% and a nearly inexistent reduction in pain-related disability. Additionally, patients in the placebo arm did not report adverse effects of the therapy [41].

It is worth noticing that participants from both groups (Belief Group and Non-Belief Group) in the two cohorts (Medical Students and Practicing Physicians) showed similar (low) knowledge about the placebo phenomenon before the experiment, even if the Practicing Physicians one was higher compared with the Medical Students. For this reason, it could be possible that the information provided to the participants, especially the ones in the Belief Group, was considered relatable and filled their knowledge gaps on placebos, hence influencing the whole experiment in a certain direction. Indeed, studies showed that humans, when faced with an emotionally intense, confusing, or ambiguous situation, try to understand it using all, albeit scarce, contextual factors available [42,43]. Our experiment is certainly limited by the number of sources of information available to participants; however, it is well recognized that clinical practice is also often characterized by ambiguous and uncertain situations [44] and it is reasonable to think that the knowledge of a physician influences therapeutic choices, just as happened in our study. In fact, during decision-making, two main factors come into play: fast and intuitive processes, which require low cognitive load, and slower and more analytic processes, which require higher cognitive engagement [45]. When physicians enter a relationship with a patient, both processes are active and intertwined, providing an overall representation of the patient, constructed form prior belief and present situations, which can shape the therapeutic process and thelater diagnosis. In fact, explicit bias and implicit bias have been found to affect physicians’ practices [45,46]. Race, ethnicity, and gender all shape clinical decision-making; for instance, it has been found that Black and Hispanic patients in emergency care received fewer analgesia treatments than white patients [47,48]. Biases have also been found toward patients’ weight [49] and age [50]. Surely, implicit bias results are hard to investigate, but of importance to take into account when analyzing physicians’ decision-making toward patients. This could be a limitation of this study, because, even if our fellow confederates (who acted as patients) were asked to give pain feedback without any facial or emotional involvement, participants could frame mainly the race and gender of their patients. Future studies should check for implicit (or explicit) bias using measures such as the Implicit Association Test (IAT) [51].

Some other limitations must be considered. First, even though this study tried to recreate a clinical situation where a physician is facing a patient, the context was hardly ecological as patients received experimental painful stimulations and were not suffering from any clinical pain condition. Also, participants involved in this study for the first group (Medical Student) were still students and far away from having the clinical experience of actual medical doctors; in fact, expertise has appeared to be crucial in different classical studies [52]. Interestingly, the second group (Practicing Physicians) showed no differences in behavior toward the role-playing patients. Future studies should investigate how different levels of expertise and knowledge can influence clinical decision-making and the administration of more or less placebo treatments, and whether they may change the therapy of a non-responding patient. Finally, patients showed no emotion during their rating to avoid bias and create a more standardized experimental condition; indeed, emotional features could change the results of this study since it has been shown that facial expressions of pain elicit empathy and adaptive behavioral responses in the observer [53]. The role of patients’ emotional manifestations on physicians’ choices should be evaluated in a controlled and standardized experimental setting.

Despite its shortcomings, this study sought to achieve new knowledge about the reasons behind the medical decision to give a placebo instead of an active treatment, and the reasons behind choosing a different therapy from the ongoing one. Both questions remain crucial in current clinical practice, particularly considering the importance of patient-centered pain management. Recent studies have shown how deception is not necessary to observe placebo effects, suggesting the prescription of open-label placebos with full transparency, evading ethical problems and paving the way for implementing placebos in clinicians’ routines [23]. Remarkably, the current study constitutes a clear frame of reference and represents the first step in the search for what defines an optimal “placebo giver”; moreover, it constitutes a novel experimental paradigm for future studies on the topic.

In general, our findings highlight the relevance of physicians’ prior knowledge about placebos and the responsiveness of the specific patient to it: the results indicate an increased probability of administering a placebo treatment only when these two sources of information are in sync. This conclusion has relevant practice implications: on one side, a proper and increased use of placebo treatments may be reached just by providing a richer education on them, and on the other side, when proper information is given, it will be the patient’s placebo responsiveness to guide physician’s decisions. The results of this study, combined with the possibility of delivering placebos with full transparency, may lead to a more ethical, efficient, and effective use in clinical settings. However, this may happen only if physicians are well-prepared for the topic. On top of this, personalized pain management may include a subpopulation that, being well responsive to placebos, receives them, evading all the side effects of active treatments.

## Data Availability

The original contributions presented in the study are included in the article, further inquiries can be directed to the corresponding author.
